# Sex-Dependent Effects of Developmental Lead Exposure on the Brain

**DOI:** 10.3389/fgene.2018.00089

**Published:** 2018-03-16

**Authors:** Garima Singh, Vikrant Singh, Marissa Sobolewski, Deborah A. Cory-Slechta, Jay S. Schneider

**Affiliations:** ^1^Department of Pathology, Anatomy and Cell Biology, Thomas Jefferson University, Philadelphia, PA, United States; ^2^Department of Environmental Medicine, University of Rochester Medical Center, Rochester, NY, United States

**Keywords:** sex, lead, developmental exposure, brain, gene, epigenetics, prenatal stress, neurotoxicity

## Abstract

The role of sex as an effect modifier of developmental lead (Pb) exposure has until recently received little attention. Lead exposure in early life can affect brain development with persisting influences on cognitive and behavioral functioning, as well as, elevated risks for developing a variety of diseases and disorders in later life. Although both sexes are affected by Pb exposure, the incidence, manifestation, and severity of outcomes appears to differ in males and females. Results from epidemiologic and animal studies indicate significant effect modification by sex, however, the results are not consistent across studies. Unfortunately, only a limited number of human epidemiological studies have included both sexes in independent outcome analyses limiting our ability to draw definitive conclusions regarding sex-differentiated outcomes. Additionally, due to various methodological differences across studies, there is still not a good mechanistic understanding of the molecular effects of lead on the brain and the factors that influence differential responses to Pb based on sex. In this review, focused on prenatal and postnatal Pb exposures in humans and animal models, we discuss current literature supporting sex differences in outcomes in response to Pb exposure and explore some of the ideas regarding potential molecular mechanisms that may contribute to sex-related differences in outcomes from developmental Pb exposure. The sex-dependent variability in outcomes from developmental Pb exposure may arise from a combination of complex factors, including, but not limited to, intrinsic sex-specific molecular/genetic mechanisms and external risk factors including sex-specific responses to environmental stressors which may act through shared epigenetic pathways to influence the genome and behavioral output.

## Introduction

Low level exposures to a variety of potential neurotoxicants can result in serious and sometimes fatal health conditions, that can appear either acutely or delayed. The incidence, manifestation, and severity of such health problems may differ between males and females (Arbuckle, 2006; Llop et al., 2013) as a result of sex-related differences in susceptibility/resilience to different environmental exposures. Unfortunately, until very recently, most basic neuroscience and epidemiological studies either excluded females or combined males and females in the analyses, limiting the appreciation of potential sex-dependent outcomes from environmental exposures.

Lead (Pb) is a potent neurotoxicant, exposure to which alters normal brain developmental trajectories resulting in an increased risk for a variety of cognitive/behavioral problems. While recent studies have documented sex-dependent effects of Pb on a variety of physiological and functional outcome measures (ex., Llop et al., 2013; Kasten-Jolly and Lawrence, 2017), the basic understanding of how sex modifies the effects of Pb on the brain are not well understood. The present review discusses some of the current ideas regarding sex-specific effects of Pb on the brain and behavior, including potential molecular mechanisms underlying sex-specific effects of Pb on the genome and epigenome. The review will first discuss findings from epidemiological and animal studies that support Pb-associated sex-specific neurobehavioral effects, followed by a brief discussion of the mechanisms involved in the sexually dimorphic organization of the mammalian brain, which if dysregulated in context of Pb exposure could partly contribute to different outcomes associated with Pb exposure. We will review some of the literature showing the interaction of Pb with environmental influences such as prenatal stress (PS) and that their interaction with sex modifies the neurotoxic effect of Pb exposure. The role of genetic variation and its interaction with sex in altering the susceptibility to Pb toxicity will also be discussed. Taken together, this review aims to highlight the extent to which epigenetic programming may be an important mechanism in determining sex-specific differential susceptibility of the brain to Pb exposure.

## Pb Exposure is a Pervasive Environmental Threat to Children

Despite the accepted fact that Pb exposure is associated with a variety of adverse health outcomes, humans continue to be exposed to Pb through a wide variety of sources including deteriorating Pb-based paint, water pipes, ceramics, herbal and cosmetic products, smelting, and other sources as well as residual contamination from years of use of leaded gasoline (Olympio et al., 2009; World Health Organization, 2010). Pb is a potent toxicant with the ability to adversely affect almost every organ in the body (World Health Organization, 2010; Flora et al., 2012), with the nervous system being particularly sensitive to its effects (Cory-Slechta, 1996; Sanders et al., 2009). The World Health Organization, in pointing out the negative long-term health effects from Pb exposure, estimated that 12.4% of the global burden of idiopathic developmental intellectual disability could be attributed to Pb exposure^1^. Thus, early life exposure to Pb remains a serious public health concern in the United States and elsewhere in the world. Children are more sensitive to, and more frequently exposed to, Pb than adults because of their behavior (ex., hand-to-mouth activities) and physiology (ex., higher percent gastrointestinal absorption) (World Health Organization, 2010). In 2012, the Centers for Disease Control and Prevention (CDC) stated that there were no levels of Pb in the blood of children that could be defined as safe and the 10 μg/dL blood Pb ‘level of concern’ was replaced with a reference value of 5 μg/dL to identify children who have been exposed to lead and who require case management. (ACCLPP, 2012). Low income, minority populations continue to be disproportionately at risk for exposure to Pb, as discussed in a recently updated CDC report (Raymond and Brown, 2017) that estimated that at least four million such households currently exist (Raymond and Brown, 2017). No obvious sex differences in blood Pb levels in children has been reported (Baghurst et al., 1992b; Stromberg et al., 2003; Vahter et al., 2007). Numerous studies, both in humans and animal models, have documented the adverse neurodevelopmental effects of Pb, however, the sex-specific effects of Pb are only now beginning to be appreciated and the mechanisms underlying these sex-dependent differences in response to Pb remain unclear.

## Lead and the Brain: Sex-Dependent Differences in Outcomes

### Prenatal Pb Exposure: Human Studies

Numerous epidemiological studies have been performed in an effort to understand the effects of Pb on the developing brain, however, a focus on sex-specific analyses of outcomes have only recently been incorporated into many of these studies. In many early epidemiological studies, sex was either not taken into account in the analysis (i.e., males and females were included but combined for analysis) or sex was included as a confounding variable along with statistical measures to eliminate the confounding effects of sex rather than examining the potential influence of sex on outcomes. In those studies where sex was taken into account in the analyses, more often than not, the neurotoxic effects appeared to be more pronounced in males than females. However, this conclusion may be misleading, as the influence of sex on outcomes may depend on the type and the age when outcomes are measured. Various statistical approaches to estimate the role of sex in modulating toxicity (such as including sex in the model, separating analysis by sex, investigating the interaction between sex and toxicant exposure, etc.) has recently been highlighted as a key aspect in the interpretation of potential sex-specific effects across studies (Buckley et al., 2017).

While detrimental neurological effects of low-level postnatal Pb exposure are well-known, effects of prenatal exposure on intellectual development have also been described. Early life deficits in sensory-motor and cognitive development have been reported based on umbilical cord blood lead levels, even at exposures below 10 μg/dL (Bellinger et al., 1984, 1986) but sex was included as a factor in the regression equation. Hu et al. (2006) reported a non-linear relationship between infant neurobehavioral performance at age 1 year and maternal Pb exposure during the first trimester of pregnancy, however, sex was included as a confounding factor in the analysis. Schnaas et al. (2006) reported that increased Pb levels in maternal blood during the third trimester of pregnancy was associated with poorer intellectual development in children, although again, the data analysis controlled for the effect of sex. In an international pooled analysis of 1,333 children followed from birth or infancy until 5–10 years of age, a strong association between intellectual impairment and low blood Pb level (<7.5 μg/dL) was reported and the sex of the child was not found to be significantly associated with the effects of Pb on IQ (Lanphear et al., 2005). In a prospective study of children in Mexico, neurodevelopment [assessed using the Bayley Scales of Infant Development II (BSID-II)] was inversely associated with infant blood Pb levels (<10 μg/dL) at 24 months age after adjusting for sex as a biological covariate (Tellez-Rojo et al., 2006). In a study (Jedrychowski et al., 2009) that analyzed cognitive outcome measures in males and females separately, male but not female neuropsychological development, as measured at age of 3 years using the Bayley Mental Development Index (MDI), was found to be inversely associated with blood Pb concentrations (>1.67 μg/dL). Another study reported a significant interaction between level of prenatal Pb exposure and neuropsychological measures of attention and visuoconstruction abilities assessed in adolescence, with males (aged 15–17 years) performing worse than females (Ris et al., 2004). A recent UK birth cohort study that investigated the association between prenatal Pb exposure (mean maternal blood Pb level 3.67 ± 1.46 μg/dL) and child IQ measured at 4 and 8 years of age showed maternal Pb exposure to have a greater effect on IQ in boys than in girls at age of 8 years or age, although the effect was statistically non-significant (Taylor et al., 2017).

In addition to adverse effects of prenatal Pb exposure on childhood cognitive development, prenatal Pb exposure has also been reported to be associated with adverse behavioral outcomes including aggression, antisocial behavior, and criminal activity (Bellinger, 2008). Wright et al. (2008) reported that prenatal Pb exposure was associated with increased arrests for violent behaviors as an adult. With each 5 μg/dL increase in blood Pb level, the number of arrests for criminal offenses reportedly increased and the mean number of arrests among males was significantly (*p* < 0.001) higher than in females. However, a recent study by Buckley et al. (2017) of 553 New Zealanders, reported that Pb exposure in childhood was only weakly associated with criminal conviction and self-reported offending from ages 15 to 38 years, in a setting where the degree of Pb exposure was not confounded by socioeconomic status. Although this study only measured blood Pb level at one time point (age 11 years), the accuracy of male sex in distinguishing between no conviction and conviction exceeded that of blood Pb level, and blood Pb level contributed a minimal increase in accuracy beyond that of male sex (Buckley et al., 2017), suggesting that the environment, living in poverty or in an unsafe neighborhood as a male, may play a large role in determining the association between blood Pb and emergent behavioral phenotypes, like criminality. Prenatal Pb exposure has also been associated with increased risk for neurodegenerative disorders in adulthood. For example, in a United States population-based study where males and females exposed to Pb as children were followed for up to 30 years, early life Pb exposure (umbilical cord blood Pb level >10 μg/dL) influenced Aß-related biological pathways associated with Alzheimer’s disease (Mazumdar et al., 2012) to a similar extent in males and females. Due to the low number of subjects in this study (*N* = 13), these results need to be viewed with caution and further work is needed to identify any potential sex-related differences in the risk of developing a neurodegenerative disorder in adulthood as a result of prenatal exposure to Pb.

Thus, while the few studies that have specifically looked at differential effects of maternal Pb exposure on male and female offspring more often than not suggest that males may be more vulnerable to the effects of Pb than females, the small number of studies and the use of different outcome measures in different studies make it difficult to draw firm conclusions regarding the modifying effects of sex on cognitive/behavioral outcomes following prenatal Pb exposures. The influence of sex on outcomes from developmental Pb exposure may very well be outcome and age dependent.

### Prenatal Pb Exposure: Animal Studies

In human epidemiological studies of prenatal Pb exposure, factors such as maternal stress and diet can modify the potential outcomes from Pb exposure in unpredictable ways. However, in animal studies, these and other variables can be explicitly controlled. Although numerous animal studies have examined sex as an effect modifier of prenatal Pb exposure, there is not a clear and consistent picture of sex-specific effects of prenatal Pb exposure. In adult mice exposed to Pb from gestational day 8 to postnatal day 21 (blood Pb level not reported), male but not female mice showed severe aggressive behavior toward their littermates as evidenced by a larger number of injured littermates in cages housing male Pb-exposed mice than in cages housing female Pb-exposed mice (Kasten-Jolly et al., 2012). The same study reported heightened anxiety, measured in an exploratory behavior assay, in female mice but not in male mice. de Souza Lisboa et al. (2005) also observed that perinatal Pb exposure (gestation/lactation) induced different behavioral alterations in adult (PND 70) male and female Wistar rats (blood Pb level > 5.0 μg/dL). Males showed increased emotionality as demonstrated by decreased rearing and increased freezing compared to females in open-field testing, while females showed increased time spent floating during the forced swimming test (de Souza Lisboa et al., 2005), an outcome that may actually be considered adaptive although associated with neural circuits underlying neurobehavioral disorders including depression (Molendijk and de Kloet, 2015). In a study of Long Evans (LE) rats exposed to Pb perinatally (gestational/lactation), only males (blood Pb level ranging from 15.38 to 28.97 μg/dL at the time of weaning) showed deficits in associative memory, assessed using a trace fear conditioning paradigm (Anderson et al., 2016). Impaired reference memory in the Morris water maze was observed in female rats with perinatal Pb exposure (Pb level measured in hippocampus, 1.73 ± 0.19 μg/g wet weight at PND21) but not in male rats (Jett et al., 1997). As the trace fear conditioning task and the reference memory component of Morris water maze task engage neural circuits differently (Morris et al., 1982; Curzon et al., 2009), the effects of Pb on these different complex functional circuits may be different in males and females. Gestational low level Pb exposure (blood Pb level ≤ 10 μg/dL) but not higher exposures (blood Pb level > 20 μg/dL), induced male-specific motor abnormalities, evidenced by decreased spontaneous motor activity and impaired rotarod performance, in adult (1 year old) mice (Leasure et al., 2008). As these examples illustrate, even in controlled animal studies, dose, timing of exposure, and specific aspects of the behavioral assessments affect the interpretation of potential sex-related differences. Also, it is important to take normal male/female differences in performance into account when trying to interpret sex-specific effects of Pb on different cognitive outcomes. For example, normal male rats tend to use the geometric configuration of the environment to navigate the Morris water maze, while females show a preference for using landmark cues to guide navigation. Thus, males and females may perform differently based on the configuration of the cues in the Morris water maze environment, as well as due to sex-specific physiological responses, such as stress responses, in this particular behavioral assay (Szuran et al., 2000; Saucier et al., 2002; Jonasson, 2005; Blokland et al., 2006; Mendez-Lupez et al., 2009). Thus, sex-specific effects of Pb exposure may need to be examined in the context of Pb-induced modifications of specific brain circuits involved in the normal sexually dimorphic expression of certain behaviors/cognitive functions. Differences in sex-dependent cognitive task performance and sex-related differences in outcomes in different behavioral tasks in Pb-exposed animals underscores the importance of including both sexes in all studies as well as including non-Pb-exposed controls, as well as multiple tests of a specific cognitive domain in order to more fully understand the implications of sex-related effects of Pb on neurodevelopment.

### Postnatal Pb Exposure: Human Studies

Although there is no “behavioral signature” *per se* to childhood Pb poisoning, even low level exposures in children can result in a spectrum of neurodevelopmental problems that include cognitive impairments (spanning decreases in verbal and performance IQs, impaired academic performance, impairments in attention, memory, and language skills) and behavioral problems (impulsivity, aggression) (Bellinger and Needleman, 2003; Canfield et al., 2003; Rogan and Ware, 2003; Bellinger, 2008; World Health Organization, 2010) that may persist into adulthood. In one of the early studies of Pb-exposed children in which sex was taken into account, a significant interaction between tooth Pb levels (5–10 μg/dL) and sex was observed, with IQ deficits more pronounced in boys (Pocock et al., 1987). In another study, a significant interaction between sex and postnatal Pb exposure was found, in which only males (15 years old) showed an inverse relationship between Pb exposure (childhood Pb level > 25 μg/dL) and attention (Ris et al., 2004). A significant inverse association between postnatal Pb exposure (difference in blood Pb level between two sexes was reported, upper range of blood Pb in boys reached 32.1 μg/dL and in girls 7.5 μg/dL) and visuomotor performance (Vermeir et al., 2005) was also found in male but not in female adolescents (assessed at approximately age 17 years). A study conducted in lead-smelting community of Port Pirie, Australia, reported that low-level Pb exposure (mean lifetime blood Pb concentration at 15 months age was 9.9 μg/dL) during early childhood was associated with decrement in neuropsychological development in both males and females at the age of 7 years (Baghurst et al., 1992a). However, females were found to be more sensitive to the effects of Pb since with increasing blood Pb concentration the decrement in full-scale IQ was more in females than in males. Two other reports from the Port Pirie Cohort Study in Australia (Burns et al., 1999; Tong et al., 2000) suggest that externalizing behavior problems such as aggressive and antisocial behavior were more frequently seen in Pb-exposed boys than in girls (lifetime mean blood Pb levels in boys and girls: 14.3 and 13.9 μg/dL) (Burns et al., 1999), whereas girls showed a greater loss of IQ points compared to boys, but this effect was not statistically significant (child blood Pb level aged 11–13 years- range between 12 and 17 μg/dL) (Tong et al., 2000). However, a recent review of three decades of the Port Pirie Cohort study suggested, among other things, that overall, there did not appear to be any age of greatest vulnerability to the effects of Pb on developmental outcomes or a threshold of effect, and at that all ages, females appeared more susceptible to Pb-associated deficits (Searle et al., 2014). These epidemiological studies of diverse populations from different countries, using various outcome measures, more often than not suggest that males might be more susceptible to Pb toxicity than females, but again, firm conclusions are limited by the small number of studies that have independently analyzed outcomes from both sexes, differences in exposures across studies, differences in outcome measures across studies, and potential confounding interactions with other uncontrollable factors. As above, this may be more of a reflection of timing of measurement and specific outcome measure.

### Postnatal Pb Exposure: Animal Studies

Relatively few studies of the effects of postnatal Pb exposures on the brain and behavior have incorporated both sexes into the study design. Chronic postnatal Pb exposure at a high concentrations (500 ppm) (blood Pb level∼70 μg/dL measured after 70 days of exposure) starting at weaning (21 days age) and lasting into adulthood (total 70 days of exposure) resulted in an anxiogenic effect observed in male but not female Swiss mice (Soeiro et al., 2007). An anxiogenic effect was also observed in adolescent male but not female mice after early postnatal Pb exposure (birth to PND15) but again, only with high level exposures (1100 ppm; blood Pb level not reported) (Ajarem et al., 2003). Disruption of olfactory recognition memory tested at PND28 after low (30 ppm) and high (330 ppm) postnatal Pb exposure from birth to PND28 (blood Pb level ranging from 0.02–20.31 μg/dL) was observed in male but not female mice (Flores-Montoya et al., 2015). Recently, it was reported that early postnatal Pb exposure (150 ppm, birth to weaning, 21 days of exposure) with blood Pb levels of 8.73 μg/dL in male and 9.67 μg/dL in female Long-Evans rats significantly impaired associative memory consolidation and recall in a trace fear conditioning paradigm in females but not in males (Anderson et al., 2016). Although this review has only mentioned a few of the studies that have described sex-related differences in outcomes from Pb exposure, what becomes apparent is that there is no clear and consistent effect that preferentially shows outcomes to be worse in one sex versus the other. As discussed earlier, understanding the influence of sex on Pb-induced neurotoxicity is complicated by the fact that different results have been obtained depending on species (rat vs. mouse) used, concentration and duration of Pb exposure, and use of different outcome measures across studies. However, what is clear is that Pb has the potential to affect males and females differently.

## Potential Molecular Mechanisms Underlying Sex-Related Differences in the Response To Pb Exposure

Data from human and animal studies unequivocally demonstrate that early life Pb exposures significantly impair cognitive, neurobehavioral, and motor functioning suggesting that these exposures alter brain structure and/or function. Both sexes are vulnerable to Pb toxicity and suffer adverse outcomes, however, evidence suggests that depending on the circumstances, one sex may be more vulnerable than the other. The underlying cause of sex differences in outcomes from Pb exposure could be linked to naturally occurring structural, neurochemical, and neuroendocrinological sex differences in the brain (Kokras and Dalla, 2014). As discussed below, the mammalian sexually dimorphic brain arises because of independent actions of, and interactions between, sex chromosomes, gonadal hormones, and epigenetic factors. The interaction of Pb with either or all of these factors, or with mechanisms linking these variables into a common framework, may differ considerably between the two sexes, could result in a sex bias in neurobehavioral or disease susceptibility to the environmental exposure. Despite the appreciation of developmentally programmed sex differences in brain structure and function, relatively few studies have attempted to investigate how these developmentally programmed sex differences in the brain might be altered by environmental exposures to Pb or what molecular mechanisms might underlie the modulation of the effects of developmental Pb exposure by sex.

### Mechanisms Underlying Sex Differences in the Brain: A Brief Survey

Understanding the pathways that influence the sexually dimorphic brain development under normal circumstances is prerequisite to understanding how disruption of these mechanisms may result in differential vulnerability/resiliency to various adverse outcomes associated with developmental Pb toxicity. The traditional model of sex differentiation in the brain, referred to as the linear serial model of sex differences, asserts that the structural/functional differences in the brain between the sexes arise due to interactions between genetic assignment of sex (XX or XY) and gonadal hormones secreted by the sexually differentiated gonads (Phoenix et al., 1959; McCarthy and Arnold, 2011). Until recently, this classic viewpoint that hormones secreted by the sexually differentiated gonads were the major driver of sex differences in the brain dominated the field. However, recent findings have challenged this perspective and it has become increasingly clear that environmental cues, integrated with gonadal sex and varying hormone levels during development (and later life) affect the network of molecular interactions that ultimately result in sex-specific phenotypes (McCarthy and Arnold, 2011; McCarthy et al., 2017a), also referred to as the ‘sexome’, the sum of sex-biased influences on gene networks and cell systems (Arnold and Lusis, 2012).

#### Influences of Sex Chromosomes: XX vs. XY

Sex chromosomes are the primary agent of sexual differentiation, with males having one X and one Y chromosome and females having two X chromosomes. The inherently different genetic content of the male and female initiates all downstream sex differences later during development. Using microarray analysis, 50 differentially expressed genes were identified in E10.5 male and female mouse brains, among which 6 were sex-linked, prior to formation of gonads, indicating that genetic factors contribute alone in sexually differentiating the brain (Dewing et al., 2003). The sex-linked genes may in turn trigger the sex-specific expression of genes on autosomes (Davies and Wilkinson, 2006), suggesting their influence on gene expression at a broader level. The genes related to X and Y chromosomes have been divided into four categories (Arnold et al., 2012; Arnold, 2017). Class I genes are Y-related genes and hence only affect males. The Y-linked *Sry* gene, an important primary sex-determining gene, leads to differentiation of the testis in males and has been characterized as a crucial sexually dimorphic gene directly affecting brain structure/functioning (Dewing et al., 2003; Czech et al., 2014; McCarthy et al., 2017b). Class II genes are X-related genes that are expressed at a higher level in XX cells (Disteche, 2012). Many of X-related genes identified in mice have been shown to play important roles in brain development and are also conserved in humans where they have been linked to intellectual disability syndromes (Ropers, 2010; Berletch et al., 2011). For example, in mice, the X-linked gene *kdm5c* plays a crucial role in brain development (Berletch et al., 2011) and in humans, *KDM5C* mutations can result in neurodevelopmental abnormalities such as autism, intellectual disability and excessive aggression in males (Goncalves et al., 2014; Brookes et al., 2015; Iwase et al., 2016). Class III genes are imprinted genes that are expressed unequally depending upon whether it is a paternal or maternal imprint on the X chromosome. XX cells receive both paternal and maternal imprints while XY cells have only a maternal X imprint. Thus, a gene that is maternally silenced would be expressed at a higher level in XX cells but not in XY cells, while a paternally imprinted gene would be more highly expressed in XY than in XX cells. Class IV genes are related to a heterochromatic X chromosome (inactivated X chromosome present in XX cells and absent in XY cells) and have been suggested to affect the expression of genes on all chromosomes indirectly (Wijchers and Festenstein, 2011). What is currently unknown is the extent to which developmental exposure to Pb affects any of these classes of X- or Y-linked genes, and if such effects exist, how they affect sex-related differences in outcomes from Pb exposure.

In recent years, X chromosome imprinted genes have received much attention as a source of sexual dimorphism in brain and their effect on brain development and functioning. Two studies by Gregg and colleagues (Gregg et al., 2010a,b), suggested that genomic imprinting in the brain varies in a brain region-, developmental stage-, and isoform specific manner that is sex-specific. For example, a significant maternal influence on gene expression was observed in the embryonic brain (E15) and a greater paternal bias was observed in the adult brain (Gregg et al., 2010b). The parental imprinting observed in the brain emphasizes the role of genomic imprinting in creating a sexually dimorphic brain that can have far reaching effects on brain function, as well as disease risk and resilience. Many studies have revealed the functional significance of parental biases in imprinting (Wang et al., 2012; Bonthuis et al., 2015; Perez et al., 2016). For example, maternal deletion, but not paternal deletion, of tyrosine hydroxylase, which is involved in regulating dopamine and norepinephrine synthesis, results in lower anxiety and anhedonia-like behaviors (Bonthuis et al., 2015). Various examples from the literature demonstrate that environmental exposures such as diet can impact imprinted gene expression patterns through epigenetic mechanisms (Gallou-Kabani and Junien, 2005) with the potential to alter behavioral programming. Depending upon how important a gene is in terms of its functional effects, sex-specific parent-of origin allelic effects can have widespread influence on brain function and, if affected by Pb exposure, could result in differences in sex specific outcomes related to Pb. Unfortunately, such studies have not yet been performed.

#### Influences of Gonadal Hormones

The sex specific action of gonadal hormones has been historically categorized into organizational and activational effects (Phoenix et al., 1959). Organizational effects are long-lasting, permanent changes induced by early exposure to gonadal hormones that differentiate males from females. The organizational effect of gonadal hormones is initiated by the release of testosterone from the developing fetal testis leading to masculinization of genitalia and other organs including brain. In the brain, the organizational effect of gonadal hormones is not limited to the prenatal period of development but continues through puberty (Schulz et al., 2009; Arnold et al., 2012; Schulz and Sisk, 2016) and is associated with brain region-specific sex differences in apoptosis, synaptogenesis and neurogenesis (for more detail see Forger et al., 2016). Activational effects are more reversible, and are associated with varying gonadal hormone levels during adulthood. Activational effects may or may not be dependent on earlier organizational effects (McCarthy et al., 2017a). Thus, effects induced by sex chromosomes and gonadal hormone secretion in utero and during postnatal development contribute in shaping the sexually dimorphic brain. These two sources of sexual differentiation, sex chromosomes and gonadal hormones, work in parallel, producing effects that can be synergistic or antagonistic, either enhancing or reducing sex differences (Arnold, 2012; Arnold et al., 2012). A recent review by Cambiasso et al. (2017) highlights the complex framework involving multiple causative factors that contribute to the structural and functional differences between male and female brains due to interactions between sex chromosomes and hormonal factors (gonadal steroids as well as steroids synthesized within the brain). Adding to the complexity of this issue, the extent to which sex chromosomes and hormones affect any sex-specific gene expression pattern in the brain is likely to be brain region-specific as well. A recent RNA seq study showed that the transcriptome of developing male and female mouse hippocampus is distinct and a subset of identified genes showed a sex-specific pattern of change through development (Bundy et al., 2017). As the hippocampus has been shown to be particularly sensitive to Pb exposure (ex., Schneider et al., 2012b; Stansfield et al., 2012; Anderson et al., 2013; Guariglia et al., 2016), it is possible that Pb may interfere with those gene expression patterns that are distinctly expressed in male and female hippocampus, potentially resulting in sex differences in hippocampal-based outcomes of Pb neurotoxicity.

#### Epigenetic Influences

Epigenetic modifications are fundamental to the regulation of biological processes and are increasingly recognized as a major contributor to the origin of sex differences in the brain through the alteration in gene expression patterns (Nugent and McCarthy, 2011; Forger et al., 2016). The findings that epigenetic changes occurring early in life have the potential to alter later life events makes them a plausible source of sustained long-term effects, including those attributed to gonadal hormones. For example, gonadal hormones have been shown to act through histone modifications (Auger et al., 2000, 2002). The binding of these hormones to nuclear receptors which then associate with the DNA directly, leads to epigenetic changes through the actions of various histone modifying enzymes, thus altering the epigenetic state (and ultimately the expression) of the gene or genes to which receptors are bound. During development, the *Sry* gene, for example, which directs testis differentiation in males, is controlled by DNA methylation in a temporal and spatially regulated manner (Nishino et al., 2004). The well-studied epigenetic modifications DNA methylation and histone modification have been linked to sexual differentiation of the brain (Nugent and McCarthy, 2011; Forger et al., 2016). Tsai et al. (2009) showed that histone methylation and acetylation levels differ by brain region in male and females even before birth (Tsai et al., 2009). Later, Matsuda et al. (2011) showed that testosterone causes sexual differentiation of H3 acetylation during early development. Through next generation sequencing methods, sex differences in the epigenetic mark, H3K4Me3, were identified across the genome in adult mouse forebrain (Shen et al., 2015). In this study, a sex bias in enrichment of H3K4Me3 wherein 71% of genes were highly enriched in females as compared to males was found. Early testosterone exposure has been shown to affect brain DNA methylation patterns in a sex-specific manner (Ghahramani et al., 2014). Recently, we also found that levels of acetylated and methylated histone modifications in the mouse brain vary dynamically throughout development (E18, PND0, PND6, and PND60) in a brain region and sex specific manner (Varma et al., 2017) and that those patterns are sensitive to early Pb exposure.

Thus, genomic and hormonal effects intricately involve or use epigenetic processes to establish sex differences in brain. Environmental factors can interact with these epigenetic mechanisms to alter the expression of genes associated with normal neurodevelopment and function potentially leading to different effects in males and females. Thus epigenomic, hormonal, and sex chromosome-related factors, acting independently or in concert with each other may be modified by environmental exposures to a neurotoxicant such as Pb, resulting in sex specific differences in outcomes from exposures. In the following sections we will briefly review some of the sex-specific molecular outcomes associated with developmental Pb exposure and suggest some possible mechanisms driving these effects.

### Interactions Between Pb, Hormones, Genome, Epigenetics, and Sex

Cecil et al. (2008) examining brains of adults who had childhood exposure to Pb, reported significant gray matter volume loss in several brain regions, with frontal cortex (FC) most affected. The inverse relationship between childhood blood lead levels and brain volume was greater in men than in women (Cecil et al., 2008). These data are particularly interesting as decreases in prefrontal gray matter volume has been associated with a predisposition to impulsive, aggressive behavior (Raine et al., 2000; Yang et al., 2005; Cannon et al., 2015), that has previously been shown to occur more frequently in Pb-exposed males than females (Bellinger, 2008; Wright et al., 2008). Sex differences in gray matter volume loss in Pb-exposed males and females might be at least partly related to effects of circulating gonadal hormones (Peper et al., 2009). Progesterone in particular may play a neuroprotective role in the brain (Seiger et al., 2016) and this hormonal influence in women exposed to Pb as children may have contributed at least in part to the less severe volumetric loss of gray matter as compared to men exposed to Pb as children. Estradiol, another sex hormone important for neurodevelopment, plays a neuroprotective role in females; moreover, estrogen receptors differ in density and distribution between males and females (Gillies and McArthur, 2010) might account for increased sensitivity in males. It has also been proposed that estrogens modify epigenetic regulatory mechanisms during critical developmental periods to induce long-lasting sex- and brain region- specific changes in neural circuits (Gagnidze et al., 2010; Nugent and McCarthy, 2011). Developmental Pb exposure during such critical windows of development could interfere with estrogen/epigenetic interactions potentially resulting in sex-specific gene expression changes in male and female brains.

**FIGURE 1:**
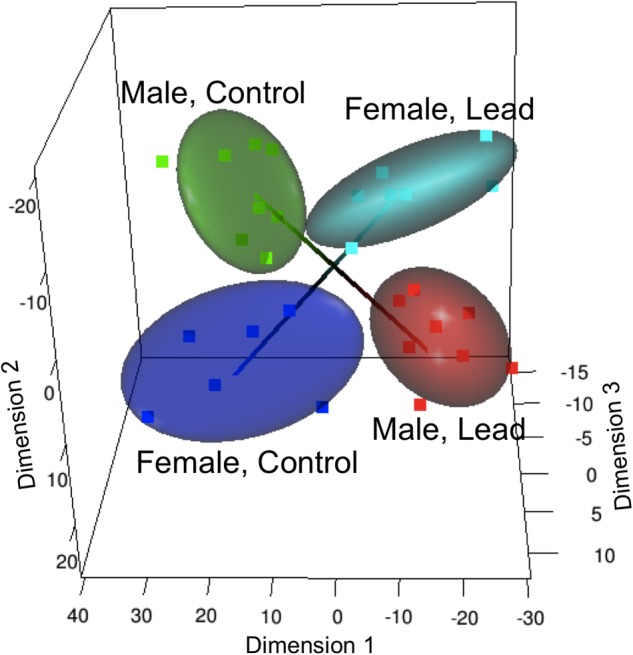
Altered sex specific transcriptome and methylome in response to Pb exposure in LE rats. Multi-dimensional scaling analysis of the gene expression response to lead (Reprinted from Schneider et al. (2011), with permission from Elsevier). Male and female groups occupy separate, diagonally opposite segments, indicating a significant sex-specific response that is in opposite directions in males and females.

Recently, studies examining Pb neurotoxicity at gene specific levels have reported sex differences in gene expression patterns that are brain-region dependent (Schneider et al., 2011, 2012b, 2013). Sex-dependent effects of Pb on gene expression, using expression microarrays, were identified in the hippocampus of LE rats following early postnatal Pb exposure (starting at weaning and continuing for 30 days) (Schneider et al., 2011). Interestingly, a group of 30 Pb responsive genes were found to be differentially expressed in opposite directions in males and females (**Figure 1**). A subset of these genes, associated with depression (Andrus et al., 2012), were significantly down-regulated in females but not in males, suggesting that females exposed to Pb might be at a greater risk at developing depression-related problems. In support of this, perinatal exposure to Pb resulted in alterations in floating time in female rats but not males (de Souza Lisboa et al., 2005), suggesting that neural circuits associated with depression in humans may be differently affected in males and females by Pb. While a study on United Sates young adults (20–39 years old) did not find sex-specific association of low level childhood blood Pb levels with major depressive disorder (Bouchard et al., 2009), females, but not males, in the Port Pirie cohort (Australia; mean childhood blood Pb concentration of 17.2 μg/dL; (McFarlane et al., 2013)) showed adult mental health problems that included social phobia, anxiety, and somatic and antisocial personality problems. Given the limitation of human studies, such as small sample size and other confounding factors, more studies are needed to conclusively determine the sex-specific differential effects of Pb exposure contributing to development of depression. Expression of genes related to transcription factors (TFs) and cyclic AMP response element binding protein (CREB), that play an important role in neurogenesis (Kandasamy et al., 2011) and plasticity were also differentially expressed in both sexes following Pb exposure (Schneider et al., 2011).

In a subsequent study, we examined the effect of perinatal and early postnatal Pb exposures on the hippocampal transcriptome in male and female LE rats (Schneider et al., 2012a). Sex, Pb exposure level, and development period of exposure modified the effects of Pb on the hippocampal transcriptome. Several important TFs that play crucial roles in brain development and function such as *Npas4*, *cFos*, *JunB*, *Egr1*, *Egr2*, and *Arc* were differentially affected in males and females. Sex-specific Pb-induced alterations in transcription factor expression can have far reaching effects on brain plasticity, since TFs are involved in regulating genes involved in diverse functional pathways (Alberini, 2009). These studies show that Pb exposure can have distinct effects on gene expression profiles in males and females and these effects seem to be dependent on exposure levels and critical windows of exposure. The sex-specific mechanism(s) underlying the distinct gene expression profiles observed in response to Pb exposure in the studies mentioned above are not clear, but may involve modulation of transcription factor interactions with sex steroid receptors leading to altered expression of target genes. Sex steroid hormone receptors such as estrogen and androgen receptors (ER and AR, respectively) can interact with TFs in an additive, synergistic or antagonistic manner influencing the target gene expression (Schule et al., 1988; Biddie and John, 2014). For example, CREB binding protein (CBP) influences ER and progesterone receptor (PR)-activated transcription influencing downstream gene expression profiles (Smith et al., 1996).

Epigenetic processes appear to be important drivers of the effects of environmental exposures on the nervous system (Hou et al., 2012; Senut et al., 2012; Marsit, 2015). One of the earliest reports suggesting a link between altered epigenome and Pb exposure comes from a study in which perturbation in global DNA methylation at Alu and LINE repeat elements in Pb exposed newborns umbilical cord blood (Pilsner et al., 2009) was reported after adjusting for sex as a confounding variable. The functional outcomes of these global perturbations remain unknown, but could possibly lead to chromosomal instability and genomic mutations. A study on adult Pb exposure reported DNA methylation induced silencing of *ALAD* promoter (*ALAD* (δ-aminolevulinic acid dehydratase) has been identified as candidate gene for Pb toxicity and is involved in Pb toxicokinetics) and down-regulation in its transcription (Li et al., 2011). Statistically significant differences in methylation frequencies between Pb-exposed and control individuals was found. The study suggested a novel mechanism of Pb toxicity through *ALAD* promoter methylation but whether epigenetic-induced *ALAD* silencing increases risk of Pb toxicity via developmental exposures in a sex-specific manner remains to be investigated. Environmental exposures such as drugs, toxicants, diet and stress may target epigenetic determinants and could result into alteration in expression of specific sets of genes or at genome wide level (Gabory et al., 2009) and this led us to examine the effects of Pb exposure on expression of chromatin modifying enzymes (Schneider et al., 2013). We observed that Pb exposure altered expression of DNMT1, DNMT3A, and MECP2 with effects that varied by sex, developmental exposure period (perinatal/early postnatal) and Pb exposure level (150, 375, and 750 ppm). An *in vitro* study examining the epigenetic effects of Pb also reported that total MECP2 and phosphorylated MECP2 at S421 protein levels were reduced in Pb exposed rat embryonic hippocampal neurons (Stansfield et al., 2012), suggesting MECP2 misregulation with Pb exposure. MECP2 is a crucial DNA methylation regulatory gene involved in regulating neuronal development and function. The association of altered MECP2 with Rett syndrome, as well as a spectrum of cognitive and behavioral disorders such as autism, mental retardation, ADHD, and mild learning disabilities (Ausio et al., 2014), warrants further investigation of role of this gene and its epigenetic modification in Pb induced neurotoxicity. Brain region- and developmental time period-specific sex differences in *Mecp2* and *Dnmt3a* expression suggest that epigenetic factors can affect spatio-temporal patterns of gene expression (Kurian et al., 2008; Westberry et al., 2010; Kolodkin and Auger, 2011). Developmental Pb exposure may further alter differential expression of *Mecp2* and *Dnmt3a* levels in males and females.

More recently, we explored Pb-induced alterations in the hippocampal methylome using promoter-based methylation microarrays and found a significant influence of sex on gene promoter methylation levels (unpublished data). We identified methylation changes in genes involved in neurobehavioral functions and neurodevelopment [for example, *PP1cb* (associated with suppression of learning and memory)], and these effects varied by sex, amount of Pb exposure, developmental window of exposure, and duration of Pb exposure. Perinatal exposure to Pb has also been reported to result in sex-specific hypermethylation in the hippocampus of female mice but not in males (Sanchez-Martin et al., 2015). Sen et al. (2015b) showed that early life Pb exposure (blood Pb level ranging from ≥ or < 5 μg/dL in Pb exposed children, ages from 3 months to 5 years, from Detroit, MI, United States) affected gene specific DNA methylation in dried blood spots in a sex-specific manner, with a greater number of CpG sites affected by Pb exposure in females. Genes showing changes in CpG methylation in females were associated with neuronal function and response to oxidative stress, and it was proposed that these methylation changes due to Pb exposure could be adaptive in females. The same group also reported effect of prenatal Pb exposure on 5-hydroxy-methyl cytosine (5hmc) in umbilical cord blood (Sen et al., 2015a) from the Early Life Exposure in Mexico to Environmental Toxicants (ELEMENT) cohort (Pilsner et al., 2009). Umbilical cord blood from male and female children were randomly selected from the 1st (<1.74 ng/dL) and 4th (>3.77 ng/dL) quartiles of blood Pb levels. They found that sex was significantly associated with Pb-induced changes in 5hmC as well as mC profiles and categorized the differentially hydroxymethylated (DhMRs) and differentially methylated (DMRs) regions into sex-independent and sex-specific differentially methylated DMRs. However, sex had a greater effect on changes in 5mC profile as compared to the 5hmC profile. The effect of Pb exposure on 5hmC is evident from the changes observed at *GSTM1* and *GSTM5* genes that had 13 and 21% decrease in hmC levels at transcription start sites, respectively. This was an interesting observation as *GST* gene polymorphisms have been associated with Pb exposure (Kim et al., 2014). The authors suggest that identified mC and hmC genomic loci could be potentially used as biomarkers of early life Pb exposure.

The information briefly reviewed above suggests that the response to Pb exposure at the level of DNA methylation may occur in a sex-specific manner and that sex-specific Pb-induced alterations in the expression of DNA methylation regulators could be important factors influencing sex-related differences in gene specific methylation levels. DNA methylation profiles in brain are sexually dimorphic (McCarthy et al., 2009; Kurian et al., 2010; Numata et al., 2012; Chatterjee et al., 2016) and hormonal influences are also believed to modify these profiles (Schwarz et al., 2010). Thus, an interaction between Pb, DNA methylation, and sex hormones may modify the brain epigenome in a manner that ultimately influences an individual’s susceptibility to the effects of Pb.

Mammalian development is a period during which dynamic changes in DNA methylation are occurring involving genomic imprinting of selected genes and thus is a vulnerable period for disruption of naturally occurring imprinting by Pb exposure, with the potential to alter developmental programs resulting in long-lasting effects. The brain is a major target of genomic imprinting and misregulation of imprinted genes in the brain following due to Pb exposure could be another plausible mechanism contributing to sex-specific neurotoxicity from Pb. Nye et al. (2016) reported that prenatal Pb exposure (median maternal blood Pb level = 0.36 μg/dL) in humans was significantly associated with DNA hypermethylation at the regulatory sequence of the autosomal *MEG3* imprinted gene, as measured in genomic DNA from umbilical cord blood. The same group also reported that while childhood exposure to Pb (mean blood Pb level between 8 and 14 μg/dL) was associated with significant DNA hypomethylation at the DMR (differentially methylated region) of the *PEG3* imprinted gene in blood of adult males and not females, neonatal Pb exposure (mean blood Pb level between 3 and 15 μg/dL) resulted in a moderate decrease in methylation at *IGF2/H19* DMR in females (Li et al., 2016). Increased methylation at the *PLAG1/HYMAI (ZAC)* DMR with neonatal Pb exposure occurred in both sexes. These data are interesting in that PEG3 is important for brain development as it regulates network of genes concerned with neural development (Li et al., 1999) and *IGF2* plays an important role in memory formation (Chen et al., 2011), implying that early Pb exposures may result in altered brain function mediated by stable DNA methylation changes at imprinted loci. Whether specific neurobehavioral outcomes are associated with misregulated imprinted genes remains to be investigated. However, characterizing the disrupted imprintome resulting from Pb exposure could potentially provide a deeper understanding of mechanisms of Pb neurotoxicity and perhaps sex-specific neurobehavioral outcomes. So far only autosomal imprinted genes have been associated with Pb exposure, but it will be interesting to know if X-linked imprinted genes are also potentially affected by Pb exposure, as well as the extent to which the influence of sex hormones and Pb exposure interact on imprinted genes through epigenetic mechanisms.

The information discussed thus far suggests that potential interactions between sex hormones and the epigenome may shape the differential response of an individual to toxic environmental exposures during development, potentially modifying the susceptibility to neurodevelopmental disorders in a sex-specific manner. Further research is required to define the roles of other epigenetic marks such as histone modifications, miRNAs and lncRNAs and the crosstalk between them, the extent to which they are altered after Pb exposure, and the extent to such Pb-induced alterations could contribute to sex-specific adverse outcomes later in life.

## Interactive Effects of Sex, Pb, and Prenatal Stress

Prenatal stress (PS) is an environmental factor that has been shown to modify the effects of Pb on the brain in numerous animal studies (ex., Cory-Slechta et al., 2004, 2008, 2010; Rossi-George et al., 2009). A recent study reported that prenatal Pb (mean maternal blood Pb level, 3.9 ± 3.0 μg/dL) and stress exposures in a human population were negatively associated with Bayley III neurodevelopmental scores in 24-month-old children and that prenatal Pb-induced neurotoxic effects were modified by PS (Tamayo et al., 2017). High levels of PS resulted in lower cognitive scores in both girls and boys at 24 months of age, but girls showed lower language scores than boys as measured by Bayley Scales of Infant Development III (BSID-III). Combined prenatal exposure to Pb and PS, but neither alone, resulted in behavioral dysfunction preferentially in female rats, measured by increased response rates on a fixed interval (FI) schedule of reinforcement (Virgolini et al., 2008). Elevated FI response rates were also associated with increased catecholamine levels in the FC. In a later study males and not females showed a trend but not a statistically significant alteration in delay discounting behavioral paradigm, which is considered to be a direct measure of impulsive choice behavior, as well as disrupted mesocorticolimbic serotonin function under Pb and PS exposure (Weston et al., 2014). The neurotransmitter serotonin which regulates activity of other neurotransmitters such as dopamine and glutamate and is associated with sex specific impulsive choice behavior, has been suggested to play a role in mediating the sex specific behavioral effects of Pb and PS (Cory-Slechta et al., 2002, 2004; Weston et al., 2014; Cowell and Wright, 2017), but more research is needed to determine how neurotransmitters affect the downstream pathways leading to sex specific behavioral outcomes in response to Pb and PS exposures. Altered corticosterone levels in male and female LE rats were also reported in response to developmental Pb ± PS exposure (Cory-Slechta et al., 2004). Pb exposure alone caused increase in corticosterone levels in adult males but elevated corticosterone levels were only observed in females following both Pb and PS exposures. The mechanisms through which altered corticosterone levels could influence glucocorticoid and mineralocorticoid receptors (GR and MR respectively), which are corticosterone dependent TFs, in a sex specific manner remains to be determined. Subtle sex differences in spatio-temporal expression of GR and MR in guinea pig brain (Owen and Matthews, 2003) have been reported during fetal and early postnatal development, suggesting that altered glucocorticoid levels during different vulnerable exposure periods for each sex may contribute to sex-related alterations in HPA axis functioning and behavior.

As both Pb and PS appear to target the epigenome (Cao-Lei et al., 2017), we have begun to investigate the extent to which altered epigenetic mechanisms may be a potential link between Pb and PS and adverse neurodevelopmental outcomes and how such effects may be modified by sex. We have examined changes in post-translational histone modifications (PTHMs) in the hippocampus (HIPP) and FC of male and female mice exposed to Pb ± PS (Pb exposure: gestation/lactation; PS: gestational). Initial studies focused on examining potential alterations in global levels of select PTHMs associated with gene expression regulation - H3K9/14Ac and H3K9Me3 (Schneider et al., 2016); H3K9Ac, H3K4Me3, H3K9Me2, and H3K27Me3 (Varma et al., 2017). Our data showed sex-, brain region-, age- and exposure- dependent differences in PTHM levels in response to Pb ± PS. For example, Pb induced a significant increase in levels of all 4 PTHMs at PND0 in female FC but not in males. Levels of all 4 PTHMs were higher at PND6 than at PND0 in male FC and were further increased by Pb+PS exposure. Pb ± PS induced variations in levels of histone marks were observed during embryonic development (E18) and extending to the neonatal stage (PND0) and persisting into adulthood (PND60), implying long lasting effects of these environmental exposures on histone modifications. An interesting observation in this study was that global levels of all 4 PTHMs varied from E18 stage to PND60 in control mice (no Pb, no PS) as well, and the levels were influenced by sex and brain-region. Interactions between sex-steroid hormone receptors and epigenetic modifiers (histone modifying enzymes) modulate transcription of target genes and influence neural sexual dimorphism (Cooke et al., 1998; Shah et al., 2004; Metzger et al., 2005; Tsai et al., 2009). Sex-dependent changes in PTHM levels in response to Pb ± PS could partly be mediated by such interactions or could be the result of other environmental factors such as behavioral experiences (Cory-Slechta et al., 2017) or genomic influences (Nugent and McCarthy, 2011). These sex-dependent changes in histone modifications may be important for normal cognitive functioning and disruption of these modifications might be linked to neurobehavioral deficits observed following Pb exposure. For example, *Kdm5c*, a histone demethylase which removes di and tri methylation of H3K4me2/3, located on X chromosome escapes X-inactivation and is expressed at a higher level in XX cells compared to XY cells. Although *Kdm5c* has a Y-paralog-*Kdm5d*, its expression level has been found not to be able to compensate for lower levels of transcripts in XY cells (Wolstenholme et al., 2013). *Kdm5c* is highly expressed in brain tissue and has been found to be expressed at higher levels in mouse brain areas such as prefrontal cortex, hippocampus and amygdala (Xu et al., 2008). Mutations in the *Kdm5c* gene has been shown to be an important cause of intellectual disability selectively in males (Stevenson et al., 2012; Goncalves et al., 2014; Brookes et al., 2015). Although the significance of dimorphic expression of *Kdm5c* in brain remains elusive, it may be an interesting candidate to investigate the extent to which its perturbation could be related to sex specific differences in outcomes associated with Pb exposure.

While the data described above have been part of a necessary first step in investigating the potential interaction between Pb, PS and the epigenome, this work needs to be followed up using unbiased genome-wide next generation sequencing methods to identify gene networks/pathways (comprising sexome, genome, and epigenome networks) that are altered by Pb ± PS exposures. A whole genome wide approach is crucial for identifying global regulators and downstream target genes affected by Pb, PS and behavioral experience, since studies to date suggest that multiple pathways and targets rather than specific single genes contribute to Pb neurotoxicity. Currently available analytic methods combined with proper bioinformatic and statistical approaches should provide important new information regarding the nature of adaptive vs. maladaptive responses to environmental exposures at the molecular level.

## Gene Polymorphism, Pb Exposure, And Sex

Other internal and external influences such as genetic background (Onalaja and Claudio, 2000) and SES (Baghurst et al., 1999; Bellinger, 2008) respectively, can potentially modify the neurotoxic effects of Pb on the brain leading to variability in neurological outcomes as seen in different epidemiological studies. However, the sex-related outcomes associated with these potential modifiers are poorly understood, except for results from a few studies which have explored the interactive effects of gene polymorphism, Pb and sex (Froehlich et al., 2007; Lamichhane et al., 2017; Zhou et al., 2017). Earlier studies have demonstrated a relation between genetic makeup and polymorphisms in a few genes (such as *ALAD* and *VDR* (vitamin D receptor) involved in Pb toxicokinetics) and Pb toxicity, but a clear protective or vulnerable role could not be linked at least through studies performed till now (Bellinger et al., 1994; Chia et al., 2004; Weuve et al., 2006; Rajan et al., 2008; Krieg et al., 2009; Gao et al., 2010; Pawlas et al., 2012; Kim et al., 2014; Schneider et al., 2014; Yun et al., 2015; Wang et al., 2017).

Froehlich et al. (2007) explored sex, *DRD4* (dopamine receptor D4 gene) and Pb exposure interactions in a study cohort where low Pb exposure (<10 μg/dL) in children has been shown to be inversely associated with IQ scores (Canfield et al., 2003) at 3 and 5 years of age. Neurobehavioral dysfunction characterized by impaired rule learning and reversal, spatial span and planning were found to be associated with increased Pb levels and were seen primarily in boys lacking the *DRD4-7* allele, suggesting that executive functioning impairment associated with Pb exposure is modified by a genetic factor × sex interaction. A recent study (Lamichhane et al., 2017) examined the relationship between maternal blood Pb exposure (<5 μg/dL), maternal *GST* (Glutathione S-Transferase) polymorphisms and sex on birth outcomes in participants in Mothers and Children’s Environmental Health (Kim et al., 2009). Previous studies on interactive effects of the *GST* gene, which plays an important role in metal-induced oxidative stress defense (Jozefczak et al., 2012), and Pb on health outcomes did not account for potential sex differences (Lee et al., 2012; Eum et al., 2013). Male infants born to mothers carrying the *GSTM1* null gene may be more susceptible to prenatal Pb exposure as indicated by three-way interaction between maternal blood Pb level, sex, and maternal *GSTM1*, in relation to head circumference at birth. A recent study in *Drosophila melanogaster* also showed a genetic variation in sensitivity to Pb exposure (Zhou et al., 2017). The study was carried out to map SNPs associated with variation in resistance to Pb toxicity in *Drosophila* and identified differentially segregating SNPs in females and males highlighting significant sex-specific effects that may determine susceptibility to Pb-induced toxicity. Functional analysis of a few candidate genes by mutations or RNAi knockdown approaches also revealed a sex-specific effect of these genes in influencing susceptibility to Pb toxicity. Interestingly, Gene Ontology (GO) analysis of the candidate genes and their human homologs were enriched for categories related to neurodevelopmental processes in both sexes. In addition, distinct GO categories were also identified in males and females. The study also identified *Drosophila* homologs of some previously characterized susceptibility genes from human studies, such as *VDR* and *GST*. Interestingly, childhood blood lead level was shown to be associated with anomalous DNA methylation at the human *PEG3* as well as *IGF2* loci (Li et al., 2016) and *Drosophila* homologs of these genes were associated with lead resistance in female flies. *Drosophila* homologs of two human genes *PAX6* and *MSl1*, which showed increased DNA methylation in Pb-exposed human ES cells (Senut et al., 2014), were also identified to be associated with Pb resistance in both sexes in flies.

The role of genetic background on modifying the effects of Pb on the male and female brain has received little attention. While there is still little known in this area, a recent study using gene expression microarray analysis in the hippocampus of males and females of three different rat strains (LE, Fischer F344, and Sprague Dawley) showed that genetic variation can influence outcomes from developmental Pb exposure (Schneider et al., 2014). The highest numbers of differentially expressed genes were identified in LE rats and the least number of differentially expressed genes were found in Sprague Dawley rats. Males and females of each strain also showed differentially enriched sets of genes.

As the effect of Pb exposure on CNS is widespread it is obvious that multiple genes and biological pathways would be involved in shaping individual responses. Hence, future research focused on genome-wide association studies in animal models such as mice (Flint and Eskin, 2012) could potentially be helpful in identifying such loci that might be relevant to Pb exposure in humans. In addition to GWAS, epigenome-wide association studies (EWAS) which are focused on identifying common variations in the epigenome and are being conducted in cancer research (Verma, 2016) to identify populations at higher risk, could also be utilized to identify variants that can explain the variation in outcomes associated with Pb exposure. Given the importance of epigenetic mechanisms in generation of Pb induced neurotoxicity it is necessary now to characterize the role of genetic variants influencing the function of epigenetic regulators such as *Dnmts*. In fact polymorphisms in *DNMTs* have been implicated in governing epigenetic susceptibility to schizophrenia in human population (Saradalekshmi et al., 2014).

**FIGURE 2:**
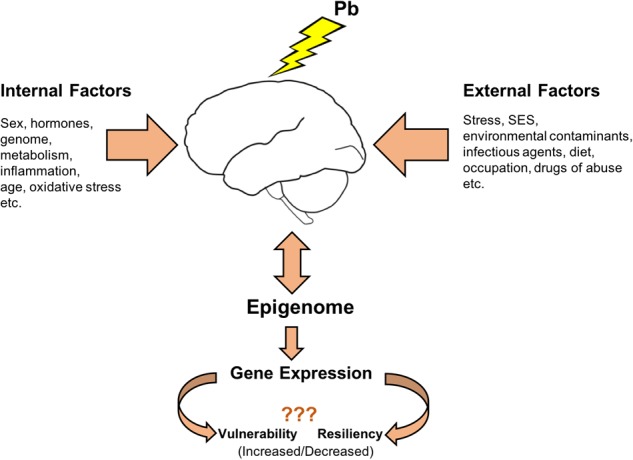
Differential susceptibility to Pb exposure: Epigenome as a key player. Pb exposure causes alterations in epigenetic processes that regulate normal gene expression patterns and modulate the brain epigenome. These epigenetic mechanisms are also shared by various internal and external factors influencing the outcomes associated with Pb neurotoxicity. A better mechanistic understanding of Pb neurotoxic effects on each sex could come from investigating how the influences from external and internal factors during the lifetime converge upon the epigenetic platform leading to different outcomes.

The term exposome was proposed to complement the genome and was described as the sum of all environmental exposures an individual experiences throughout life (Wild, 2005). Later the term was redefined (Miller and Jones, 2014) to include cumulative biological responses to exposures from environment, diet, behavior and endogenous processes (epigenetic alterations, DNA mutations and protein modifications). The term exposome is truly applicable to the study of effects from Pb exposure wherein adaptive as well as maladaptive responses to Pb need to take into account other environmental and endogenous influences occurring throughout one’s life to ultimately influence outcomes. Considering sex-specific responses to Pb exposure, it is appropriate to expand the term exposome to include the variable of sex, requiring that sex be considered as biological variable in all studies of Pb toxicity. With accumulating evidence that epigenetic mechanisms are targeted by Pb exposure and that these epigenetic processes are also well integrated with other endogenous and external influences, understanding epigenetic programming may be the key to understanding differential susceptibility to Pb exposure (**Figure 2**).

## Conclusion

This review has highlighted some of the known sex-related outcomes associated with early life exposure to Pb and some of the potentially different mechanisms involved in males and females. The review has also highlighted that it is becoming increasingly clear that multiple factors can further influence an individual’s response to Pb and that epigenetic factors, as targets of Pb toxicity, may play key role in programming the long-term effects from early life Pb exposure. While much remains unclear regarding the sex-specific responses of the brain to Pb, one thing that is very clear is that much more study is necessary in order to further identify and understand the complex mechanisms contributing to different outcomes from Pb exposure in males and females (**Box 1**). The molecular networks involved in defining the sexome, genome and epigenome are integrated with each other and the need now is to identify those factors that interact with these biological entities and integrate them into a mechanistic framework to understand the ways in which sex is an effect modifier for Pb neurotoxicity.

**Box 1. Questions and Challenges.**

(1) Are there global regulators/factors at the molecular level that play a critical role in determining adaptive/maladaptive responses to Pb?
(2) If such factors exist, how are they modulated by sex, genetic background, and other environmental exposures?
(3) Can such factors occupy important nodes in gene networks to integrate multifactorial influences so that even small magnitude changes, in aggregate, may result in significant functional effects?
(4) Integration of current unbiased genome wide technologies with appropriate bioinformatic approaches and statistical designs are needed in order to unravel these complex multifactorial mechanisms of biological responses to toxic exposures.

## Author Contributions

GS wrote the initial draft of the manuscript and worked on subsequent revisions. All the other authors worked on revising the manuscript.

## Conflict of Interest Statement

The authors declare that the research was conducted in the absence of any commercial or financial relationships that could be construed as a potential conflict of interest.

## Footnotes

1 http://www.who.int/mediacentre/factsheets/fs379/en/
